# Very low mutation burden is a feature of inflamed recurrent glioblastomas responsive to cancer immunotherapy

**DOI:** 10.1038/s41467-020-20469-6

**Published:** 2021-01-13

**Authors:** Matthias Gromeier, Michael C. Brown, Gao Zhang, Xiang Lin, Yeqing Chen, Zhi Wei, Nike Beaubier, Hai Yan, Yiping He, Annick Desjardins, James E. Herndon, Frederick S. Varn, Roel G. Verhaak, Junfei Zhao, Dani P. Bolognesi, Allan H. Friedman, Henry S. Friedman, Frances McSherry, Andrea M. Muscat, Eric S. Lipp, Smita K. Nair, Mustafa Khasraw, Katherine B. Peters, Dina Randazzo, John H. Sampson, Roger E. McLendon, Darell D. Bigner, David M. Ashley

**Affiliations:** 1 Department of Neurosurgery, Duke University Medical Center, Durham, NC 27710 USA; 2 The Preston Robert Tisch Brain Tumor Center at Duke University Medical Center, Durham, NC USA; 3 Department of Computer Science at the New Jersey Institute of Technology, Newark, NJ 07102 USA; 4 Tempus Labs, Inc., Chicago, IL 60654 USA; 5 Department of Pathology, Duke University Medical Center, Durham, NC 27710 USA; 6 Department of Biostatistics, Duke University Medical Center, Durham, NC 27710 USA; 7 The Jackson Laboratory for Genomic Medicine, Farmington, CT 06032 USA; 8 Department of Systems Biology at Columbia University, New York, NY 10032 USA; 9 Department of Surgery, Duke University Medical Center, Durham, NC 27710 USA; 10 Istari Oncology Inc., Morrisville, NC 27560 USA; 11 School of Medicine, Deakin University, Geelong, VIC Australia

**Keywords:** CNS cancer, Immunoediting, Cancer immunotherapy

## Abstract

Several immunotherapy clinical trials in recurrent glioblastoma have reported long-term survival benefits in 10–20% of patients. Here we perform genomic analysis of tumor tissue from recurrent WHO grade IV glioblastoma patients acquired prior to immunotherapy intervention. We report that very low tumor mutation burden is associated with longer survival after recombinant polio virotherapy or after immune checkpoint blockade in recurrent glioblastoma patients. A relationship between tumor mutation burden and survival is not observed in cohorts of immunotherapy naïve newly diagnosed or recurrent glioblastoma patients. Transcriptomic analyses reveal an inverse relationship between tumor mutation burden and enrichment of inflammatory gene signatures in cohorts of recurrent, but not newly diagnosed glioblastoma tumors, implying that a relationship between tumor mutation burden and tumor-intrinsic inflammation evolves upon recurrence.

## Abstract (web summary)

Recurrent glioblastomas (rGBM) have dismal outcomes, but long-term survival has been observed in subsets of patients after immunotherapy. Here the authors report a positive association between low tumor mutation burden, inflammatory gene signatures, and survival after immunotherapy in rGBM patients.

## Introduction

Despite aggressive standard of care (SOC) therapy, recurrence of WHO grade IV malignant glioma (glioblastoma, GBM) is virtually inevitable. No effective salvage therapy exists for recurrent GBM (rGBM), for which median survival is ~12 months. A subset of rGBM patients exhibited durable long-term survival^1^ following intratumor infusion of a recombinant rhino:poliovirus hybrid, PVSRIPO^2^. PVSRIPO kills neoplastic cells and induces innate inflammation that primes anti-tumor T cells^3^. Similarly, subsets of rGBM patients have responded to other virotherapy approaches^4^ or immune checkpoint blockade (ICB)^5^.

Such dichotomous responses have been observed in other indications after ICB therapy^6^, inspiring investigation into predictive biomarkers of ICB response^7^. High tumor mutation burden (TMB) is associated with response to ICB in several cancer types, with gliomas among the noted exceptions^8^. Rather, some hypermutant gliomas with mismatch repair (MMR) deficiency are less responsive to PD1 blockade than gliomas with lower TMB^9^, and a non-significant inverse relationship between TMB and radiographic/histological responses to PD1 blockade was observed in an rGBM patient cohort^10^. Using clinical and genomic information from three immunotherapy clinical trial cohorts^1,8,10^ (Table 1), we sought to define common features of the subsets of immunotherapy-responsive rGBM patients.

**Table 1:** Overview of data sets used in this study.

	Immunotherapy-treated cohorts						Comparison cohorts					
Cohort:	PVSRIPO rGBM		Anti-PD1/PD-L1 GBM				TCGA pGBM		GLASS rGBM		Wang rGBM	
Reference:	Desjardins et al.^1^		Samstein et al.^8^		Zhao et al.^10^		N/A		Barthel et al.^11^		Wang et al.^16^	
Information used for this study	Survival, TMB, RNAseq		Survival, TMB		Survival, TMB, RNAseq		Survival, TMB, RNAseq		Survival, TMB,		Survival, TMB, RNAseq	
Comparison	*n*	Fig.	*n*	Fig.	*n*	Fig.	*n*	Fig.	*n*	Fig.	*n*	Fig.
Pre-Tx TMB vs. survival	21	1a, S1	81	1c	29	1d	277	1e	132	1f	–	–
IDH1 wt	19	1b	76	1c	21	1d	–	–	–	–	–	–
Exclude *MGMT* meth:	14	S2a	–	–	–	–	–	–	–	–	–	–
IDH1 wt; no *MGMT* meth:	13	S2b	–	–	–	–	–	–	–	–	–	–
PTEN wt only:	17	S2j	–	–	–	–	–	–	–	–	–	–
TTR vs. survival	45	1b	–	–	–	–	–	–	131^a^	1g	–	–
IDH1 wt:	35	S2d	–	–	–	–	–	–	–	–	–	–
Exclude *MGMT* meth:	28	S2e	–	–	–	–	–	–	–	–	–	–
IDH1 wt; no *MGMT* meth:	22	S2f	–	–	–	–	–	–	–	–	–	–
*TP53* mut vs. survival:	34	S2c	–	–	–	–	–	–	–	–	–	–
IDH1 wt:	31	S2g	–	–	–	–	–	–	–	–	–	–
Exclude *MGMT* meth:	22	S2h	–	–	–	–	–	–	–	–	–	–
IDH1 wt; no *MGMT* meth:	21	S2i	–	–	–	–	–	–	–	–	–	–
Steroids vs. survival (TMB cohort):	21	S2k	–	–	–	–	–	–	–	–	–	–
Steroids vs. survival (TTR cohort):	45	S2j	–	–	–	–	–	–	–	–	–	–
Inflammatory gene sets vs. TMB:	14	2b, S4-6	–	–	11	2b, S5,6	193	2, S5,6	–	–	25	2b, S5,6
Immunoediting vs. TMB:	18^b^	2c	–	–	–	–	–	–	115^c^	2d	–	–
Paired pGBM vs. rGBM	–	–	–	–	–	–	–	–	–	–	25	2e/f, S8

*Tx* treatment, *TTR* time to first recurrence, *meth* methylation.

^a^Only tumors tested at first recurrence.

^b^Tumors sequenced on the same WES panel.

^c^Neoantigen (neo-Ag) ratios available from a subset of the cohort.

Here we show that rGBM patients carrying low TMB survive longer after polio virotherapy and ICB. rGBM tumors with lower TMB have enriched inflammatory gene signatures relative to rGBM tumors with higher TMB levels. This correlation is not observed in primary GBM tumors, indicating that a relationship between tumor-intrinsic inflammation and TMB develops upon recurrence in GBM.

## Results

### Very low TMB identifies rGBM patients with longer survival after immunotherapy

**Fig. 1:**
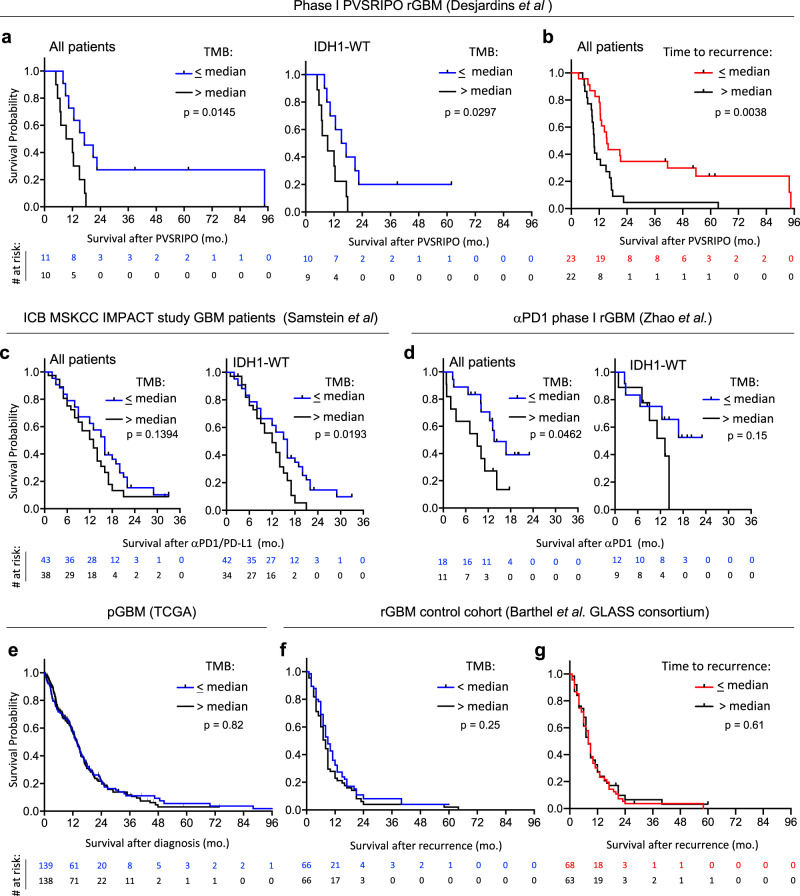
Very low TMB identifies rGBM patients with longer survival after immunotherapy. **a**, **b** Phase-1 PVSRIPO cohort patient survival stratified by median TMB (1.3 mutations/Mb) (**a**) or median time to first recurrence (11.02 months) (**b**). **c**, **d** Survival after ICB administration in patients stratified by cohort median TMB (median TMB: 3.94 for **c**; 1.04 and 0.767, respectively, for the two merged cohorts in **d**, see methods for dichotomization strategy explanation). **e**–**g** Survival of immunotherapy-naïve primary (**e**; median = 1.5 mutations/Mb) or recurrent (**f**; median = 3.14 mutations/Mb) GBM patients stratified by cohort median TMB; or time to recurrence after initial diagnosis of GBM (**g**). **a**–**g** All *p* values are from Log-rank Mantel-Cox test comparing survival between strata (two-tailed).

We determined that rGBM tumors from patients with longer survival (>20 months) after PVSRIPO treatment harbored very low TMB (<0.6 mutations/Mb, Supplementary Fig 1, *n* = 21). Stratifying patient survival after PVSRIPO treatment by median TMB (1.3 mutations/Mb) confirmed longer survival of rGBM patients carrying ≤ median TMB (Fig. 1a); of note only 2/21 patients in the > median TMB strata were hypermutated (>10 mutations/Mb). TMB was previously shown to correlate with time to recurrence from initial surgery^11^, and a lack of non-synonymous mutations in *TP53* (ref. ^12^). Accordingly, shorter time to first recurrence (Fig. 1b, preceding PVSRIPO treatment, *n* = 45) and lack of *TP53* mutation (Supplementary Fig. 2, *n* = 34) also identified patients with longer survival after PVSRIPO therapy. Survival differences upon stratification by TMB, time to recurrence, and *TP53* mutation status were maintained after excluding patients with IDH1 mutation and/or *MGMT* promoter methylation (Supplementary Fig. 2); survival differences upon stratification by TMB were also observed after exclusion of patients with PTEN mutations (Supplementary Fig. 2k). Suggesting these findings are not related to steroid dosing, stratification of cumulative pretreatment dose of steroids (2 days prior to, and the day of, treatment) did not reveal survival differences (Supplementary Fig. 2k, l).

To test if very low TMB associates with longer survival after ICB in rGBM patients, we compared survival after treatment with ICB in two cohorts stratified by TMB^8,10^. Patients with ≤ median TMB lived longer after receiving ICB therapy than those with >median TMB in both cohorts (Fig. 1c (*n* = 81; 3 hypermutants total), d (*n* = 29; no hypermutants)). We confirmed that stratification of post-PVSRIPO/ICB survival by TMB remained significant following exclusion of patients with hypermutation, indicating these differences were not driven by unfavorable responses in hypermutated patients (Supplementary Fig. 3). Very low TMB is associated with favorable survival in WHO grade III anaplastic astrocytoma, but not in WHO grade IV GBM^9,13^. Indeed, we confirmed no survival differences in immunotherapy-naïve primary (*n* = 277) or rGBM (*n* = 132)^11^ patient cohorts upon stratification by TMB (Fig. 1e, f), or by time to first recurrence (Fig. 1g, *n* = 131). Together these findings imply that increased survival of immunotherapy-treated GBM patients with very low TMB is due to immunotherapy response.

### Tumor immune status inversely associates with TMB in rGBM tumors

**Fig. 2:**
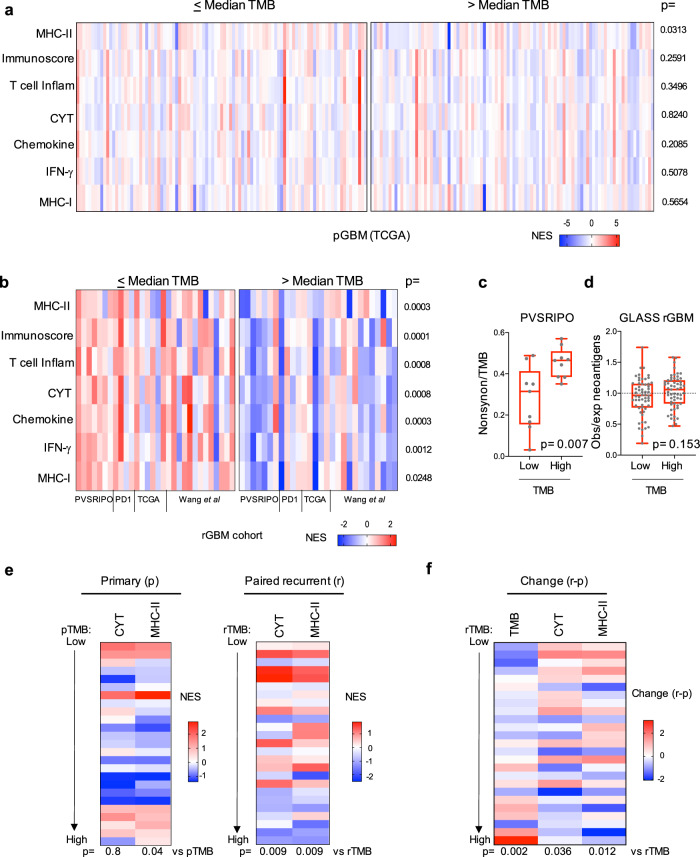
Tumor immune status inversely associates with TMB in rGBM tumors. **a**, **b** ssGSEA values dichotomized by respective cohort median TMB are shown for the pGBM TCGA (**a**; median = 1.65 mutations/Mb) or merged data from four rGBM cohorts after stratification by cohort median TMB (**b**; 1.3 (PVSRIPO cohort), 1.8 (PD1), 3.6 (TCGA), or 3.3 (Wang et al.) mutations/Mb); *p* values are from Mann–Whitney test (two-tailed). **c**, **d** Median TMB stratification of the ratio of: nonsynonymous mutation/Mb to TMB for PVSRIPO cohort analyzed on the same WES panel (**c**; median = 1.2 mutations/Mb), or expected/observed neoantigen ratios for the GLASS cohort (**d**; median = 3.55 mutations/Mb); box represents quartiles and median, and whiskers indicate range; *p* values are from unpaired Mann–Whitney test (two-tailed); **c** *n* = 18 and **d** *n* = 116 patients. **e** Paired RNA-seq data were analyzed by ssGSEA for cytolytic/MHC-II signatures for primary (left) vs. recurrent (right) tumors and ordered by TMB. **f** Change in TMB or ssGSEA values (CYT and MHC-II) was determined by subtracting primary values from corresponding recurrent values for each patient (“r–p”). Change in ssGSEA (r–p) values were plotted by increasing recurrent TMB (“rTMB”), Spearman *p*-values are shown below heatmap. NES = normalized enrichment score.

High TMB associates with inflammatory profiles in select tumor types, potentially explaining its link with favorable response to ICB therapy^14^. Inflammation-associated gene expression measured via single sample gene set enrichment analysis (ssGSEA)^15^ revealed minimal differences in tumors with TMB below vs. above median in IDH1-wildtype (wt) primary GBM (pGBM, TCGA) patients (Fig. 2a; *n* = 193). To test this in rGBM, we sequenced RNA from all available pre-treatment tumor biopsy tissue samples in the PVSRIPO cohort (Supplementary Fig. 4, *n* = 14, all IDH1-wt); we also tested other previously sequenced IDH1-wt rGBM cohorts (refs. ^10,16^, and TCGA). In contrast to IDH1-wt pGBM, stratification of IDH1-wt rGBM ssGSEA scores by median TMB revealed markedly higher inflammation in rGBM tumors with lower TMB (Fig. 2b, Supplementary Fig. 5, *n* = 58); this correlation was also evident along a continuum (Supplementary Fig. 6).

Neoantigen depletion/immunoediting may explain a relationship between low TMB and immune activity. The ratio of nonsynonymous mutations to total TMB was lower in tumors with lower overall TMB in the PVSRIPO-treated cohort (Fig. 2c; *n* = 18). Likewise, in a larger cohort of rGBM tumors^11^, the ratios of observed vs. computationally expected neoantigens (Fig. 2d; *n* = 115) were non-significantly lower in tumors carrying lower overall TMB; this trend was exclusive to rGBM (Supplementary Fig. 7). Thus, rGBM tumors with very low TMB are immunologically engaged and may be immunoedited; these relationships were not apparent in primary, treatment-naïve GBM.

To determine how an association between TMB and immunological activity emerges in rGBM, we analyzed TMB and transcriptome immune signatures from paired primary and recurrent IDH1wt GBM tumors (Fig. 2e, f; *n* = 25 pairs, all <10 mutations/Mb)^16^. As observed for pGBM tumors from TCGA (Fig. 2a), there was limited correlation between TMB and inflammation-associated ssGSEA scores in the primary tumors; yet a correlation was observed in the recurrent tumors from the same patients (Fig. 2e and Supplementary Fig. 8). Calculating the change in these features upon recurrence revealed enrichment in cytolytic score (CYT) and MHC-class II (MHC-II) gene sets in patients with relatively lower TMB at recurrence (rTMB; Fig. 2f). Decreases in TMB were also primarily observed in patients with very low rTMB. Thus, enrichment of inflammatory signatures upon GBM recurrence—concurrent with TMB suppression—may explain the relationship between very low TMB and immune status of rGBM. Notably, CYT and MHC-II gene enrichment scores were quantitatively similar between primary and recurrent tumors at a cohort level, despite heterogenous changes at the patient level (Supplementary Fig. 8d, e). Together these observations indicate that a relationship between TMB and immune status evolves upon recurrence.

## Discussion

Our findings, in conjunction with recent studies in hypermutant rGBM patients^9,17^, suggest utility of TMB to predict immunotherapy outcome in rGBM. Notably our study included only a handful of hypermutant patients that did not drive the differences observed upon stratification by TMB; rather relative to non-hypermutant rGBM patients with moderate TMB levels, patients with very low TMB survived longer after immunotherapy. ICB and PVSRIPO immunotherapy engage antitumor T cell immunity^3^. Immunologically active tumors are a feature of ICB-responsive patients in other indications^14^, in some cases in patients with low TMB^18^, and very low TMB associates with increased tumor-intrinsic inflammation in rGBM (Fig. 2). Thus, we postulate that the baseline inflammatory status of rGBM tumors determines their susceptibility to immunotherapy. Correlation between tumor immune status and TMB was robust in rGBM, but not pGBM, suggesting that this relationship is a consequence of SOC therapy.

Since neoantigen depletion was also observed in rGBM patients with very low TMB, a possible explanation for the link between TMB and tumor immune status is that mutation levels, the landscape of which evolve during/after SOC therapy^19^, are restricted in a subset of patients with active and/or functional immune surveillance. Nonetheless, low TMB tumors are equally lethal to their high TMB counterparts in immunotherapy naïve patients, indicating that despite such potential immune engagement/immune-surveillance, immune evasion still occurs. Thus, it is possible that tumors with low TMB, which are more likely to be inflamed in rGBM patients, may employ different strategies to evade immune surveillance relative to their higher TMB counterparts, and that the immune evasion mechanisms of inflamed rGBM tumors may be successfully targeted by immunotherapy. Possibly suggesting an immune subversive mechanism: low TMB also associated with higher MDSC/myeloid cell density within tumors (Supplementary Fig. 4c, d), cell types well-known for suppressing effector functions of antitumor T cells^20^—including via the PD1/PD-L1 axis^21–23^—that are also potentially reprogrammed by PVSRIPO therapy^3,24^. Alternatively, as a shorter time to recurrence also correlates with lower TMB and favorable response to PVSRIPO, shorter duration of SOC, or shorter time from initial surgery, may allow for superior response to immunotherapy. In support of the latter, surgery has been shown to bolster efficacy of neoadjuvant αPD1 therapy^25^.

Thus, TMB itself may not be a causative driver of response to immunotherapy/tumor inflammation, but rather may merely reflect the immunological status of tumors or one of several other potential co-related features. Such features may include time to recurrence (and extent of tumor evolution), *TP53* mutation, as well as any differences in the clinical care between patients with high vs. low TMB (e.g. differences in long-term corticosteroid exposure). Our study is insufficient to identify a specific number or threshold of TMB that predicts immunotherapy response in rGBM patients, but reveals an unexpected correlation between TMB, tumor-intrinsic inflammation, and survival after immunotherapy in rGBM patients that may hold predictive biomarker potential upon further validation/investigation. Future studies are needed to determine how SOC therapy alters the tumor immune status of GBM, and how reliably TMB, as well as potential TMB-related features, predicts response to immunotherapy in rGBM patients.

## Methods

### Patient selection

For the PVSRIPO cohort (Duke University), patient tumor biopsy specimens, obtained within 24 h prior to intratumor infusion of PVSRIPO, were acquired from a completed dose finding and toxicity study of PVSRIPO in rGBM (NCT01491893), the results of which have been previously reported^1^. Patients provided written informed consent for the conduct of these studies under an IRB approved protocol at Duke University. All patients with sufficient tissue for whole-exome sequencing (WES) were analyzed for this study, resulting in a cohort of 21 subjects from which tissue was acquired on the day of PVSRIPO infusion; 34 were sequenced at any time point after recurrence, including from autopsy (used to determine *TP53* status). Relevant patient demographics, as well as survival information (updated as of April 29, 2020), are presented in Supplementary Table 1. Additional de-identified cohorts from previously published studies include: the MSKCC IMPACT study containing GBM patients treated with either αPD1 or αPD-L1 (in two cases with combined αCTLA4)^8^, αPD1-treated rGBM cohort (Zhao et al.^10^)^18^, the Glioma Longitudinal AnalySIS (GLASS) consortium^11^, Wang et al.^16^, and TCGA.

### Study design

This study was approved by the Duke Institutional Review Board and acquired de-identified samples linked to patient clinical information and demographics, via a sample-specific unique ID number. All patients were consented to these analyses upon clinical trial enrollment. For the PVSRIPO cohort, WES and RNA-seq were performed by TEMPUS, Inc. and computational analyses were conducted jointly by TEMPUS, Inc., researchers at Duke University, and the New Jersey Institute of Technology.

### Nucleic acid extraction, library preparation, and sequencing

For the PVSRIPO cohort, germline DNA was extracted from blood collected in a PAXGene blood tube. Total nucleic acid was extracted from formalin-fixed paraffin-embedded (FFPE) tumor tissue sections that were microdissected (if deemed necessary by pathologist prediction of tumor cellularity) and digested with proteinase K. Total nucleic acid was extracted with a Chemagic360 instrument from both blood and tumor samples using a source-specific magnetic bead protocol. Total nucleic acid was the input for all DNA library construction; RNA was purified from total nucleic acid via DNaseI digestion followed by magnetic bead purification. Nucleic acid quantification was performed by a Quant-iT picogreen dsDNA reagent Kit or Quant-iT Ribogreen RNA Kit (Life Technologies). Quality was tested using a LabChip GX Touch HT Genomic DNA Reagent Kit or LabChip RNA High HT Pico Sensitivity Reagent Kit (PerkinElmer). Sequencing (WES and RNAseq) was performed on an illumina Hi-Seq 4000 system using patterned flow cell technology at TEMPUS, Inc.

### TMB assessment, mutational analysis, and patient cohort stratification

For the PVSRIPO cohort, tumor-specific mutations were calculated via the JANE workflow orchestration tool (Tempus Labs, Inc.). FASTQ files were analyzed using FASTQC for quality control assessment, and aligned with Novoalign (Novocraft, Inc.) with integrated adapter trimming turned on. Following alignment, the SAM files were converted to BAM files, sorted, and duplicates were marked. Following alignment and sorting, a set of quality control steps were run on the BAM files to ensure data quality was sufficient for high sensitivity variant calling. Subsequent to quality control, variants were called with both tumor and normal samples in joint mode to generate VCF files. Callable regions were obtained by GATK3Callable Loci with default parameters. TMB was calculated as total variants divided by the total callable bases covered by the WES panel in megabases (Mb) for both intronic and exonic sequence (total variants/Mb) or for exonic and splice site sequence (nonsynonymous mutations/Mb). Only variants with an allelic fraction of at least 10% were used for TMB calculation. Previously computed coverage-adjusted TMB values associated with pGBM TCGA samples were downloaded from tcia.at^26^. All relevant information from the MSKCC IMPACT study^8^ was downloaded from cbioportal.org^27,28^, including coverage-adjusted TMB values and survival information. Coverage-adjusted TMB values from rGBM TCGA, GLASS consortium, and the Wang et al. cohort were previously determined and provided by study authors^11,16^. In the αPD1 data set, previously computed coverage-adjusted TMB values for tumors sequenced at Columbia University were provided by the study authors^10^; for a subset of samples sequenced by Foundation Medicine, Inc. (FMI), the number of mutated genes and number of genes covered by sequencing were provided by the study authors and used to compute nonsynonymous mutations/Mb^10^. Since different methods were used to compute the two cohorts in the Zhao et al. αPD1 data set, Columbia WES and FMI cohorts were first separately stratified by their respective cohort median TMB and then merged together as ≤ or > median TMB for data in Fig. 1d. Only patients sequenced at Columbia University with available matched RNA-seq data were used in the αPD1 cohort presented in Fig. 2. For all cohorts: all stratifications by median were performed within each distinct cohort to mitigate influence of different sequencing panels/approaches and TMB calculation methodologies. For consistency, in instances where a specimen’s TMB or time to recurrence value equaled the cohort median value (i.e. with odd specimen number, or when multiple specimens shared the median value—relevant to the αPD1/PD-L1 cohorts, Fig. 1c, d), ≤ median was used. Median values/stratification values are indicated in the associated raw data supplement.

### RNA-seq data processing and analysis

RNA-seq data were aligned to GRCh38 using STAR (2.4.0.1) and expression quantification per gene was computed with FeatureCounts (1.4.6). RNA-seq count data normalized to TPM (transcripts per kilobase million) values were used as input for ssGSEA. ssGSEA was performed for select signatures using the GSVA package^29^ with default settings. The association of each signature’s ssGSEA scores with TMB was tested using the Spearman correlation test; raw ssGSEA values were centered and scaled for heatmap presentation. Analyses determining change in ssGSEA values were derived via subtracting primary from recurrent ssGSEA values for each gene set followed by normalization. ssGSEA gene sets chosen were chosen based upon their relevance to ICB responsive tumors^14^, and Reactome/Biocarta database analyses.

### Statistics

In survival analyses: for patients who were alive at the time of analysis, survival time was censored at the date of last follow-up. The Kaplan–Meier estimator was used to describe the distribution of survival time; the Log-rank Mantel-Cox test was used to compare survival between different strata. Tests used for other analyses in Fig. 2 are presented in the figure legend. Statistical analyses were performed using SAS software, version 9.4 (SAS Institute) or GraphPad Prism version 8; *p*-values are denoted in figures. For survival analyses in the MSKCC and GLASS cohorts, a few patients had post-treatment/recurrence survival values of “0” and were excluded form plots/analyses as indicated in the associated supplemental raw data spreadsheet. All data points and statistical analyses represent individual patients; Spearman *p*-values are two-tailed. All box and whisker plots denote median + quartiles (box) and range (whiskers).

### Reporting summary

Further information on research design is available in the Nature Research Reporting Summary linked to this article.

## Supplementary information

Supplementary Information

Reporting Summary

## Data Availability

The whole-exome sequencing and RNA-seq data associated with the PVSRIPO clinical trial cohort are available at dbGAP accession code phs002270.v1.p1. Raw data associated with other published cohorts have been shared; details are in their respective manuscripts; and raw data are available publicly as follows: “Wang et al” EGAS00001001033^16^ and EGAS00001001041^16^; “Samstein et al” cohort (https://www.cbioportal.org/study/summary?id=glioma_msk_2018)^8^; “Zhao et al” cohort (https://www.cbioportal.org/study/summary?id=gbm_columbia_2019)^10^; GLASS consortium cohort (http://synapse.org/glass)^11^; and TCGA (https://portal.gdc.cancer.gov). A reporting summary for this article is available as a Supplemental Information file. The remaining data are available within the Article, Supplemental Information or are available from the authors upon request. Source data are provided with this paper.

## Source data

Source Data
