# Supplementary material for: Very low mutation burden is a feature of inflamed recurrent glioblastomas responsive to cancer immunotherapy
Source: Nat Commun. 2021 Jan 13;12:352. doi: 10.1038/s41467-020-20469-6 (PMC7806846; doi:10.1038/s41467-020-20469-6)
Supplement: Supplementary file 1 — Supplementary Information [file 41467_2020_20469_MOESM1_ESM.docx]

**Supplementary Information**

Low tumor mutation burden is a feature of inflamed recurrent glioblastomas responsive to cancer immunotherapy

Gromeier and Brown *et al*

**Supplementary Figure 1.** Genomic features of PVSRIPO Phase 1 clinical trial cohort patient tumors. Top panel: GBM-associated and DNA damage response genes were assessed for mutation/deletion. Middle panel: patients were ordered by post-PVSRIPO treatment survival (as of April 29, 2020; data bars shown in grey/black). Bottom panel: Total variants (black bars) or nonsynonymous mutations/splice site mutations (green bars) were plotted. Additional published cohorts of glioma patients included in this study are previously described: Zhao *et al* 2019 describing correlates of response to PD1 blockade in rGBM patients, Barthel *et al* 2019 and the GLASS consortium, Wang *et al* 2017, and TCGA (See Materials and Methods for further description and citations).

**Supplementary Figure 2.** Analysis of potential genomic and clinical confounding features for relevance to low TMB and survival after PVSRIPO. PVSRIPO cohort patients were stratified by cohort median TMB, time to recurrence, or *TP53* mutation followed by exclusion of patients with: *MGMT* promoter methylation (**a**, **e**, **h**), IDH1 mutation (**d**, **g**), or either IDH1 mutation or *MGMT* promoter methylation (**b**, **f**, **i**). (**c**) Survival of PVSRIPO cohort patients with and without coding mutations in *TP53*. (**j**) Stratification of PVSRIPO cohort patients by TMB after removal of patients with PTEN mutations. (**k, l**) Stratification of survival of n=21 TMB (k) or n=55 time to recurrence (i) PVSRIPO cohorts by median cumulative steroid dose in the two days preceding, and day of PVSRIPO clinical administration. (**a-i**) All p values are from two-tailed Log-rank Mantel-Cox test.

**Supplementary Figure 3.** Survival of PVSRIPO (a) or αPD1/PDL1 (b) treated cohorts stratified by median TMB after removal of patients with hypermutated TMB levels (>10 mutations/Mb); p values are from two-tailed Log-rank Mantel-Cox test.

**Supplementary Figure 4.** Characteristics of the PVSRIPO RNA-seq cohort and relevant RNA-seq based analyses. (**a, b**) Comparison of post-PVSRIPO survival (**a**) and TMB (**b**) between patients analyzed by RNA-seq versus all patients on which TMB information is available immediately prior to PVSRIPO therapy, survival updated as of April 29, 2020. (**c**) Computational prediction of various immunological cell types using ssGSEA. (**d**) Spearman R values for correlation of each cell type from (c) with TMB is plotted, asterisks denote p< 0.05. Exact p values for significant Spearman R correlation in (d) are as follows: iDC p= 1.27x10^-6^, Macrophages p=1.04x10^-5^, Tfh cells p= 5.13x10^-5^, Mast cells p=6.69x10^-5^, B cells p= 0.00044, T cells p= 0.001, MDSC p= 0.001, Tcm cells p=0.008, NK CD56bright cells p=0.01, Neutrophils p=0.011, NK CD56dim cells p=0.011, DC p=0.0158, Th1 cells p= 0.0158, Eosinophils p=0.0397, aDC 0.0476, Treg cells p=0.031, Th2 cells p=0.0036, and T helper cells 0.003.

**Supplementary Figure 5.** Violin plots of normalized ssGSEA enrichment scores for above (grey) vs below (violet) median cohort for IDH1 wt pGBM (**a**, corresponding to Fig 2a) and IDH1 wt rGBM (**b**, corresponding to Fig 2b). P values are from two-tailed Mann-Whitney test.

**Supplementary Figure 6.** Very low TMB is associated with inflammatory gene expression profiles in rGBM cohorts. (**a**) Normalized ssGSEA scores ranked by increasing TMB for the pGBM TCGA cohort (n=193). (**b**) Normalized ssGSEA scores ranked by increasing TMB for the PVSRIPO cohort (n= 14), Zhao *et al* PD1 cohort (n= 8), recurrent GBM tumors from TCGA (n= 11), and the Wang *et al* rGBM cohort (n=25). (**c**) Samples with *MGMT* promoter methylation were excluded from PVSRIPO cohort ssGSEA data. (**d**) Spearman R values (two tailed) are provided for each gene set within each cohort; exact p values are as follows: PVSRIPO cohort: CYT p=0.0018, Immunoscore p= 1x10^-6^, MHC-cII p= 5.7x10^-8^; Wang rGBM cohort: CYT p=0.0085, T cell inflam p=0.041, Immunoscore p= 0.0329, MHC-cII p= 0.0089; and PVSRIPO cohort no MGMT: MHC-cI 0.049, IFN-g p= 0.0047, Chemokine p= 0.0016, CYT p= 0.0058, T cell-inflam p= 0.0003, Immunoscore p= 0.002, MHC-II p= 0.0002.

**Supplementary Figure 7.** Evidence of immunoediting in glioma patients by TMB. All cohorts are from the GLASS consortium; in each context cohorts were separated by low (below cohort median) TMB or high (above cohort median) TMB. (**a**) Neoantigens/nonsynonymous ratios for rGBM tumors (median TMB= 3.432 mutations/Mb, n=144). (**b**) Observed/expected neoantigen ratios for primary GBM tumors (median TMB= 2.84 mutations/Mb, n=114). (**c**) Observed/expected neoantigen ratios for recurrent anaplastic astrocytoma tumors (median TMB= 2.4 mutations/Mb, n=52). (**d**) Observed/expected neoantigen ratios for primary anaplastic astrocytoma tumors (median TMB= 1.6 mutations/Mb, n=62). Box represents quartiles, center line represents median, and whiskers indicate minimum and maximum values. Unpaired t-test p values are from two-tailed test.

**Supplementary Figure 8.** Genomic changes associated with recurrence in GBM patients. Paired RNA-seq data was analyzed for TMB or ssGSEA enrichment scores; p= primary, r= recurrent. (**a**) side-by-side TMB/ssGSEA enrichment scores for each patient ordered by TMB of recurrent tumor. (**b**) Change in TMB/ssGSEA enrichment scores was determined by subtracting primary TMB/ssGSEA values from recurrent values for each patient. (**c**) Spearman R for change in TMB/ssGSEA from (b) correlated to TMB at recurrence (rTMB, left panel; TMB p=0.0019, CYT p=0.0362, MHC-II p=0.0122) or change in TMB (right panel; MHC-I p=0.044); asterisk indicates p< 0.05. (**d**) Paired CYT and MHC-II scores enrichment scores for p and rGBM tumors. (**e**) Paired CYT and MHC-II scores separated by < or > cohort median rTMB (3.33 mutations/Mb); red= increase, blue= decrease.
