# Supplementary material for: Very low mutation burden is a feature of inflamed recurrent glioblastomas responsive to cancer immunotherapy
Source: Nat Commun. 2021 Jan 13;12:352. doi: 10.1038/s41467-020-20469-6 (PMC7806846; doi:10.1038/s41467-020-20469-6)
Supplement: Supplementary file 2 — Reporting Summary [file 41467_2020_20469_MOESM2_ESM.pdf]

nature research

Corresponding author(s): David Ashley

Last updated by author(s): Oct 13, 2020

Reporting Summary

Nature Research wishes to improve the reproducibility of the work that we publish. This form provides structure for consistency and transparency in reporting. For further information on Nature Research policies, see our Editorial Policies and the Editorial Policy Checklist.

Statistics

For all statistical analyses, confirm that the following items are present in the figure legend, table legend, main text, or Methods section.

n/a	Confirmed	
☐	☒	The exact sample size (*n*) for each experimental group/condition, given as a discrete number and unit of measurement
☐	☒	A statement on whether measurements were taken from distinct samples or whether the same sample was measured repeatedly
☐	☒	The statistical test(s) used AND whether they are one- or two-sided<br>*Only common tests should be described solely by name; describe more complex techniques in the Methods section.*
☐	☒	A description of all covariates tested
☒	☐	A description of any assumptions or corrections, such as tests of normality and adjustment for multiple comparisons
☐	☒	A full description of the statistical parameters including central tendency (e.g. means) or other basic estimates (e.g. regression coefficient) AND variation (e.g. standard deviation) or associated estimates of uncertainty (e.g. confidence intervals)
☐	☒	For null hypothesis testing, the test statistic (e.g. *F*, *t*, *r*) with confidence intervals, effect sizes, degrees of freedom and *P* value noted<br>*Give P values as exact values whenever suitable.*
☒	☐	For Bayesian analysis, information on the choice of priors and Markov chain Monte Carlo settings
☒	☐	For hierarchical and complex designs, identification of the appropriate level for tests and full reporting of outcomes
☐	☒	Estimates of effect sizes (e.g. Cohen's *d*, Pearson's *r*), indicating how they were calculated

Our web collection on statistics for biologists contains articles on many of the points above.

Software and code

Policy information about availability of computer code

Data collection	DNA and RNA sequencing data were generated using Illumina HiSeq 4000. Raw data from publicly available data sets were downloaded from TCGA or cBioportal.org.
Data analysis	Novoalign (Novocraft, Inc; v3.08), GATK CallableLoci (v3) , STAR (v2.4.0.1) (alignment of RNA-seq data), FASTQC (v0.11.8), JANE workflow orchestration tool (Tempus Labs, proprietary), Feature Counts (v1.4.6), GSVA package (reference #29, v1.35.7), Microsoft Excel (v16.41), GraphPad Prism (v8), and SAS (v9.4).

For manuscripts utilizing custom algorithms or software that are central to the research but not yet described in published literature, software must be made available to editors and reviewers. We strongly encourage code deposition in a community repository (e.g. GitHub). See the Nature Research guidelines for submitting code & software for further information.

Data

Policy information about availability of data

All manuscripts must include a data availability statement. This statement should provide the following information, where applicable:

- Accession codes, unique identifiers, or web links for publicly available datasets
- A list of figures that have associated raw data
- A description of any restrictions on data availability

The data associated with the PVSRIPO cohort has been deposited in dbGAP under accession code (phs002270.v1.p1). Data from other publicly available cohorts are accessible at repositories indicated in their respective published studies and as follows: "Wang et al" EGAS00001001033 (https://www.ebi.ac.uk/ega/studies/EGAS00001001033) and EGAS00001002429 (https://www.ebi.ac.uk/ega/studies/EGAS00001002429); Samstein et al cohort (https://www.cbioportal.org/study/summary?id=glioma_msk_2018); Zhou et al cohort (https://www.cbioportal.org/study/summary?id=gbm_columbia_2019); GLASS consortium cohort (http://synapse.org/glass); and TCGA (https://portal.gdc.cancer.gov). All plotted values/data to construct figures in this manuscript are available in the Source Data file

provided with the manuscript.

Field-specific reporting

Please select the one below that is the best fit for your research. If you are not sure, read the appropriate sections before making your selection.

☒ Life sciences ☐ Behavioural & social sciences ☐ Ecological, evolutionary & environmental sciences

For a reference copy of the document with all sections, see nature.com/documents/nr-reporting-summary-flat.pdf

Life sciences study design

All studies must disclose on these points even when the disclosure is negative.

Sample size	All patients with available tumor and blood/saliva (germline) material for sequencing was used for the phase I PVSRIPO cohort. All publicly available cohorts with information relevant to our studies were analyzed here to our knowledge.
Data exclusions	For neoantigen depletion values associated with the GLASS cohort (values were acquired from reference 11 study authors; Fig 2d), 3 specimens harboring less than 3 total neoantigens were excluded to prevent artifacts of determining the absolute neoantigen:silent mutation ratios with such small absolute numbers; this exclusion was consistent with data quality measures used in the study from which they were derived (reference 11). In Fig 1c (MSKCC IMPACT study) two patients (one in <median and one in >above median) had OS values of "0" and were excluded as they could not be plotted. Likewise in Fig 1f and g (GLASS cohort), 4 patients in <median, and 6 patients in > median TMB strata were excluded due to post recurrence survival values of "0".
Replication	We confirmed that low TMB associates with longer survival after PD1/PDL1/ICB in two distinct cohorts and in PVSRIPO treated patients; these are the only publicly available immunotherapy treated GBM cohorts with matched genomic features that we are aware of. We also tested the association of TMB with inflammatory transcriptome signatures in all publicly available GBM cohorts/patients we are aware of. This included 4 distinct rGBM cohorts where these associations were all observed independently (Fig 2b extended data fig 5), pGBM from two cohorts (Fig 2a&e; extended data fig 5), as well as matched primary and recurrent GBM specimens (Fig 2e). Key genomic analyses for the PVSRIPO cohort were analyzed redundantly by separate investigators: TMB values were calculated by 3 different computational teams at Tempus Labs, Duke University, and New Jersey Institute of Technology with consistent results; the correlation of higher inflammatory transcriptome features in patients with low TMB in the PVSRIPO cohort was confirmed by computational experts at both Tempus Labs and New Jersey Institute of Technology independently.
Randomization	For TMB (Fig 1 and 2) stratifications each cohort was individually stratified by the cohort respective median TMB to mitigate the influence of different sequencing panels/methods on TMB calculation. Notably, within the Zhao et al anti-PD1 cohort (Fig 1d) two different sub-cohorts were included that were sequenced on different panels; thus within this cohort the two sub-cohorts were first stratified by median TMB, and then merged together as above or below the respective cohort median. For consistency, in instances where a specimen's TMB or time to recurrence value equaled the cohort median value (i.e. with odd specimen number in the cohort, or when multiple specimens shared the median value- relevant to the anti-PD1/PD-L1 cohorts, Fig 1c, d), the additional numbers were included in the below median strata. Median values/stratification values are indicated in the associated raw data supplement.
Blinding	For the Duke PVSRIPO cohort, computational calculations of TMB and ssGSEA were performed blinded of clinical outcome.

Reporting for specific materials, systems and methods

We require information from authors about some types of materials, experimental systems and methods used in many studies. Here, indicate whether each material, system or method listed is relevant to your study. If you are not sure if a list item applies to your research, read the appropriate section before selecting a response.

Materials & experimental systems

n/a	Involved in the study
☒	☐ Antibodies
☒	☐ Eukaryotic cell lines
☒	☐ Palaeontology and archaeology
☒	☐ Animals and other organisms
☐	☒ Human research participants
☐	☒ Clinical data
☒	☐ Dual use research of concern

Methods

n/a	Involved in the study
☒	☐ ChIP-seq
☒	☐ Flow cytometry
☒	☐ MRI-based neuroimaging

Human research participants

Policy information about studies involving human research participants

Population characteristics	For the PVSRIPO cohort (Duke University), patient tumor biopsy specimens, obtained within 24h prior to intratumor infusion of PVSRIPO, were acquired from a completed dose finding and toxicity study of PVSRIPO in rGBM (NCT01491893), the results of which have been previously reported (reference #1). Relevant patient demographics, as well as survival information (updated as of April 29, 2020), are presented in Extended Data Table 1. Additional de-identified cohorts from previously published studies include: the MSKCC IMPACT study containing GBM patients treated with either aPD1 or aPD-L1 (in two cases with combined aCTLA4; reference #8), aPD1-treated rGBM cohort (Zhao et al 2019; reference #17), the Glioma Longitudinal AnalySIS (GLASS) consortium (reference #11), Wang et al 2017 (reference #16), and TCGA.
Recruitment	For the PVSRIPO cohort, patients with sufficient tissue for whole exome sequencing (WES) were analyzed for this study, resulting in a cohort of 21 subjects from which tissue was acquired on the day of PVSRIPO infusion; 34 were sequenced at any time point after recurrence, including from autopsy (used to determine TP53 status). No selection bias is anticipated as these analyses are retrospective. We acquired de-identified samples linked to patient clinical information and demographics, via a sample-specific unique ID number. All patients were consented to these analyses upon enrollment in the phase I PVSRIPO clinical trial. All other cohorts were previously published and disclosed methods of study enrollment, consent, etc.
Ethics oversight	This study was approved by the Duke Institutional Review Board

Note that full information on the approval of the study protocol must also be provided in the manuscript.

Clinical data

Policy information about clinical studies

All manuscripts should comply with the ICMJEguidelines for publication of clinical research and a completedCONSORT checklist must be included with all submissions.

Clinical trial registration	NCT01491893
Study protocol	The full clinical trial protocol and associated study analyses have been previously published (reference #1).
Data collection	Data were collected at Duke University beginning in 2012 as described in reference #1. Clinical follow-up/survival information were updated as of April 29th, 2020 for this study.
Outcomes	Primary and secondary endpoint results of the clinical trial itself were previously published (reference #1). For the current study, updated survival or time of last follow-up was used to compare to genomic features and time to recurrence.
